# Supplementary material for: Rapid Generation and Molecular Docking Analysis of Single-Chain Fragment Variable (scFv) Antibody Selected by Ribosome Display Targeting Cholecystokinin B Receptor (CCK-BR) for Reduction of Chronic Neuropathic Pain
Source: Int J Mol Sci. 2023 Jul 3;24(13):11035. doi: 10.3390/ijms241311035 (PMC10341521; doi:10.3390/ijms241311035)
Supplement: Supplementary file 1 [file ijms-24-11035-s001.zip › ijms-2404363-supplementary.pdf]

Supplementary Materials

1. Plasmids: Strains and Reagents

All reagents used in the study were commercially available and were of reagent grade or better. All restriction enzymes and DNA modification enzymes were of molecular biology grade. All primers were purchased from Invitrogen and Integrated DNA Technologies (IDT). pGEM-T easy cloning vector and TNT T7 Quick for PCR DNA kit (rabbit reticulocyte cell free extract) were purchased from Promega. Rosetta-gami™ (DE3) Competent Cells and pET32a Plasmid were purchased from Novagen (USA). HRP conjugated, mouse anti-his antibody was purchased from GenScript Inc.

A peptide (Biotin-CETPRIRGTGTRELE) corresponding to the extracellular N-terminus of mouse CCK-BR (residues 39–53) was synthesized with biotin at the N-terminus by Genscript (USA) (**Scheme S1**). Peptide purity (≥95%) and sequence were verified by high-performance liquid chromatography and mass spectrometry (**Schemes S2 &S3**).

1.1. Immunization of Mice

BALB/c mice were obtained from The Charles River. Mice (n = 5) were immunized with a 15-amino-acid extracellular CCKB receptor peptide (MW 2142.21, >95.4%, sequence CETPRIRGTGTRELE with a biotin tagged N-terminal, in N-methyl-2-pyrrolidone solvent) and inflammation was confirmed with a positive ELISA [32,33]. Splenocytes of individual vaccinated mice were harvested 6 wk following inflammation and were placed in 10 ml of TRIzol for use in RNA isolation [31].

1.2. Rapid Generation of Single Chain Antibodies by Ribosome Display

1.2.1. Antibody Library Construction

Total spleen RNA was prepared as described by Kunamneni et al. [32,33]. Mice spleens were minced and homogenised in 10 mL TRIzol (Invitrogen). The total RNA pellet was air-dried and resuspended in 500 µL of nuclease-free water (stored at −80 °C). Complementary DNA (cDNA) was synthesized from approximately 25 µg of total RNA using a SuperScript *VILO Master Mix* (Invitrogen, USA) following the manufacture's instructions provided.

For antibody library construction, the PCR primers were based on published sequences [32,33,67] with minor modifications (**Table S1**). The primers were designed to introduce in-frame NcoI and NotI restriction sites to the 5′ end of the VH sequence and to the 3′ end of the VL sequence, respectively. The VH_F/VH_R and VL_F/VL_R sets of primers (**Table S1**) were used for PCR amplification of VH and VL gene segments using the cDNA template. The VH_R and VL_F set of primers (**Table S1**) were used to introduce overlapping sequences which enabled the scFv gene fragments to be assembled by overlap extension PCR and these primers encode a 20 amino acid linker sequence $(G_4S)_4$. The amplified heavy and light-chain products were purified and pooled, and an aliquot of light and heavy-chain templates was subjected to overlap extension PCR amplification using Link to introduce Kozak sequence on the 5′ end and an overlap extension on the 3′ end to facilitate joining to the variable heavy-chain libraries using MVKR and RDT7. Finally, the PCR product encoding all the variable heavy-chain and light-chain combinations was amplified with primers RDT7 and MVKR to introduce T7 site into Kz-conjugated VH-VL library and to produce the DNA encoding the anti-CCK-B immunoglobulin scFv libraries. The initial PCR amplification reactions were performed at a 52 °C annealing temperature with 30 cycles, and the subsequent library assembly step used 16 cycles with Q5® High-Fidelity 2X Master Mix (NEB, USA) and 20 pmol of each primer pair per reaction. DNA fragments were resolved by gel electrophoresis on 1% (wt/vol) agarose gels. DNA isolation from agarose gels was carried out following QIAquick DNA gel purification kit instructions. The final purified PCR product ~0.8 kb is a template for Ribosome Display.

1.3. Cell-free Ribosome Display Technology

To select specific antibody fragments, we have used our modified eukaryotic ribosome display as described previously [32,33,68]. In vitro transcription and translation reaction was based on a coupled rabbit reticulocyte lysate system (Promega's TNT quick-coupled transcription-translation system) and performed according to the supplier's protocol. The PCR-generated DNA library of antibody-coding genes derived from the spleens of five mice were expressed in this lysate system. Briefly, 50 μL of transcription/translation mixture containing 40 μL of TNT T7 Quick Master Mix, 2 μL of DNA library (0.1 to 1.0 μg), 1 μL (1 mM) of methionine, 1 μL of DNA enhancer, and 6 μL of water were added, and the reaction mixture was incubated at 30°C for 90 min. Then, 5 μL of RNase-free DNase I (Roche) (2000 U/mL) was added, and the mixture was incubated for 20 min at 30 °C (in order to remove the DNA template so that subsequent PCR only picks up pulled down RNA sequences).

1.4. Selection of CCK-BR scFvs

To select specific antibody fragments, the streptavidin plate wells were coated with 1 μg/mL of the CCK-B peptide in 100 μL PBS at 4 °C overnight. Protein coated wells were washed with PBS and blocked with 100 μL of molecular biology grade Bovine Serum Albumin (BSA) in PBS (10 mg/mL) (New England Biolabs) for 1 h at room temperature. The translation/transcription mixture [containing the protein-ribosome-mRNA (PRM) complexes] was added to the washed and blocked protein-coated tubes and incubated on ice for 1 h. The PCR tubes were washed three times with ribosome display washing buffer (PBS containing 0.01% Tween 20, 5 mM Mg acetate and 0.1% BSA, pH 7.4) and two times quick wash with ice-cold RNase-free water, and the retained RNA (antibody sequences) subjected to the following recovery process; in situ Single-Primer RT-PCR Recovery was performed in the PCR tubes carrying selected ARM complexes using a SuperScript *VILO Master Mix* (Invitrogen, USA). The obtained cDNA was amplified in a 25 μL PCR *Q5* High-Fidelity 2X *Master Mix* for 35 cycles of 30 s at 94 °C, 30 s at 65 °C, and 1 min at 72 °C, and 10 min at 72 °C with MVLR and RDT7 using Q5 master mix (NEB, USA). The RT-PCR product from a single round of ribosome display was purified by agarose gel electrophoresis. The purified PCR products were used for the next round of ribosome display.

1.5. Cloning, Expression and Purification of an Anti-CCK-B scFvs

The RT-PCR product from a third round of ribosome display was cloned into the pGEM®-T Easy vector (T-Cloning® Kit, Promega) according to the manufacturer's instructions [32,33]. The ligation products were transformed in pGEM®-T Easy vector *E. coli* cells (XL1-Blue) and positive colonies (~100) were chosen randomly by blue-white selection and confirmed by DNA sequencing using T7 and SP6 standard primers (**Table S1**). Based on the sequencing results, the clones in right reading frame without stop codon were chosen for prokaryotic expression.

For the construct of scFvs, DNA was amplified with the forward primer, RDT7-5′CTATAGAAGGGTAATACGACTCACTATAGGGCGAATTCCACCATGGCC3′ (with an NcoI restriction sequence highlighted in bold) and the reverse primer, MVLR-5′AGT**GCGGCCGC**ATCAGCCCGTTTTATTTCCAA3′ (with an NotI restriction sequence highlighted in bold) [32,33]. Both set of primers allow these two amplicons to be subcloned into a pET32a-His vector and were transformed into Rosetta Gami (DE3) *E. coli* strain and plated onto LB agar plate with 100 μg/mL of carbenicillin, and grown at 37 °C for 16 h. The molecular weight and isoelectric points were predicted using ExPASy bioinformatics resource portal (http://web.expasy.org/compute_pi/) [69]. Next day, five colonies were inoculated in 3 mL of LB media with the same antibiotics and grown at 37 °C with 225 rpm shaking for 16–18 h and the positive clones were selected by restriction analysis with NcoI and NotI and confirmed by DNA sequencing. The 10 mL overnight culture was used to prepare a glycerol bacterial stock and inoculated into 200 mL of LB medium containing the same antibiotics and grown at 37 °C with shaking at 225 rpm until OD_{600} reached between 0.4–0.6. The culture was briefly chilled on ice to 25 °C and the cells were induced by the addition of IPTG (final concentration 1 mM) and were incubated for 12 h at 25 °C with shaking. Cells were harvested in 4 × 50 mL tubes by centrifugation at 4000× *g*, 4 °C for 20 min. Cell pellets were frozen and stored at −80 °C before undergoing further processing.

The 50 mL cell pellet from 200 mL culture was re-suspended in 3 mL of lysis buffer (20 mM Tris–HCl, 500 mM NaCl, 20 mM Imidazole, 0.1 % Triton X-100 pH 8.0). Cells were lysed by sonication on ice (6 × 30 s) and were centrifuged at 14,000× *g* for 15 min to remove cellular debris. The soluble fraction was filtered through 0.2 μm filters and applied to HisTrap excel 1ml column (GE Life Sciences, USA). The column was equilibrated and washed with 20 mM Tris–HCl, 500 mM NaCl, 20 mM Imidazole pH 8.0 and the sample was eluted 20 mM Tris–HCl, 500 mM NaCl, 500 mM Imidazole, pH 8.0 in one elution step. The purification was carried out at a constant flow of 1 mL/min. Fractions of 1 mL were collected through the elution step. The purified protein fractions were concentrated using a Millipore 10K Concentrator and kept at 4 °C. The pooled fractions were resolved by size exclusion chromatography in 25 mM Tris, pH 7.4, 150 mM NaCl, 0.02% NaN_3. The purified protein was filtered through 0.2 μm filter and aliquoted in a biosafety cabinet. The purified protein was analyzed by SDS-PAGE, Western blot, SE-UPLC and LAL method.

1.5.1. Sodium Dodecyl Sulfate Polyacrylamide Gel Electrophoresis (SDS-PAGE) Analysis

The prepared proteins were analyzed using SDS-PAGE. All the samples were mixed with 2× sample loading dye (0.2% bromophenol blue, 4% SDS, 20% glycerol, 100 mM Tris-HCl, pH 6.8) containing 100 mM dithiothreitol (DTT) for reduction. For the preparation of non-reducing samples, DTT was excluded. The samples were subjected to 12% SDS-PAGE gel after boiling at 100 °C for 10 min. After electrophoresis, the gels were stained with Coomassie brilliant blue (0.25% Coomassie blue, 50% ethanol, 10% acetic acid) and destained (50% ethanol, 10% acetic acid). The gels were imaged and visualized using ChemiDoc XRS+ System (Bio-Rad, Hercules, CA, USA).

1.6. Western blot of scFv fragments.

The proteins (20 μg) were separated by SDS-PAGE and were blotted onto polyvinylidene difluoride (PVDF) membrane using an iBlot2 Gel TransferDevice (Life Technologies) for 7 min [32,33]. The membranes were blocked in 5% w/v skimmed milk powder/TBS buffer at room temperature for 1 h, then incubated with mouse anti-His antibody (GenScript, Piscataway, NJ, USA) at a dilution of 1:5000 for 1 h. After three washes with TBST buffer, HRP-conjugated Donkey anti-mouse antibody (diluted 3/10,000 in TBST) (Abcam, USA) at room temperature for 1 h, followed by washing with TBS-T buffer 3× for 10 min each. The protein was detected and visualized with Amersham ECL detection reagent (GE Healthcare, USA) following the manufacturer's recommendation.

1.8. Protein Aggregation Analysis by SE-UPLC

2 u L of sample (1 mg/mL) was injected into ACQUITY UPLC Protein BEH SEC 200, 1.7 μm, 4.6 × 150 mm column with a flow of 0.3 mL/min for 10 minutes. A mobile phase of 50 mM Sodium Phosphate, 500 mM NaCl, pH 6.2 was used. Size-based antibody separation was performed on an SEC column with UV detection. Soluble aggregates by area percent (if present) were detected and measured.

1.9. Octet RED384 Kinetic Measurements

scFv77-2 antibody prepared at 5 μg/mL in 1× KB (PBS pH [7.4], 0.02% Tween-20, 0.1% albumin, and 0.05% sodium azide) running buffer was dispensed into a 384-well tilted-bottom microplate at a volume of 90 μL per well. Each concentration occupied 8 vertical wells. A second 384-well microplate containing mouse CCK-B peptide at 7 titrated concentrations (250 nM–1.03 nM, in 3-fold serial dilutions), the glycine [pH 1.5] regeneration solution, and 1× KB buffer for baseline stabilization was also prepared. Both plates were agitated at 1000 rpm over the entire course of the experiment. A total of 8 HIS1K (penta-his antibody capture) sensor tips were used for a scFv (8 sensors) per binding cycle. Prior to the binding measurements, the sensor tips were pre-hydrated in 1× KB for 5 min, followed by 3 cycles of pre-conditioning with 15-s dips in glycine (pH 1.5), alternating with 30-s dips in 1× KB. The sensor tips were then transferred to the scFv-containing wells for a 180-s loading step. After a 30-s baseline dip in 1× KB, the binding kinetics were measured by dipping the scFv-coated sensors into the wells containing mouse CCK-B peptide at varying concentrations. The binding interactions were monitored over a 240-s association period and followed by a 420-s dissociation period in new wells containing fresh 1× KB buffer. All measurements were corrected for baseline drift by subtracting a

control sensor exposed to running buffer only. Data were analyzed using a 1:1 interaction model (fitting global, Rmax unlinked by sensor) on the ForteBio data analysis software Octet Analysis Studio software 12.2.0.20.

2. Endotoxin assay

To measure the endotoxin levels, a Toxin Sensor Chromogenic LAL Endotoxin Assay Kit (Cat. No. L00350, Genscript, USA) was employed following the manufacturer's instructions. CCK-B scFv was diluted with endotoxin-free water to adjust the concentration to 1 µg/mL. A 100 µL volume of a 1 µg/mL sample, or an endotoxin standard (1, 0.1, 0.05, 0.025, 0.01 EU/mL), was dispensed into an endotoxin-free vial, and 100 µL of LAL reagent was added, mixed, and incubated at 37 °C for 50 min. After incubation, 100 µL of chromogenic substrate solution was added to each vial, mixed, and incubated at 37 °C for 6 min. A 500 µL volume of stop solution (color-stabilizer #1) was added to each vial and mixed, followed by 500 µL of color-stabilizer #2. Finally, 500 µL of color-stabilizer #3 was added to each vial and mixed thoroughly for 3 s. After transferring 150 µL of the reaction mixture into each well of a 96-well plate, a SpectraMax iD5 microplate reader (Molecular Devices, USA) was used to measure the absorbance at 545 nm. After analyzing the absorbance, the units of endotoxin were calculated using a standard curve obtained from standard solutions.

Supplementary Figures

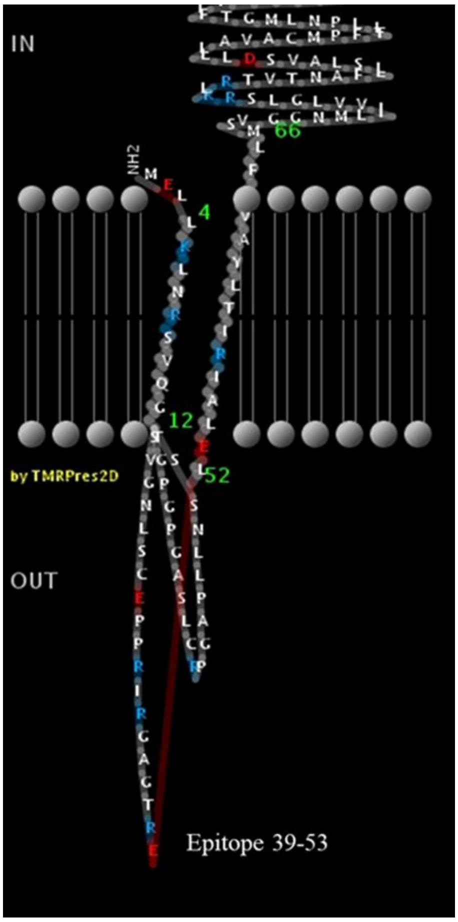

Scheme S1A. The amino acid sequence of mouse Gastrin/cholecystokinin type B receptor and a predicted model of B-barrel outer membrane domain. A two-dimensional (2D) figure of the predicted CCK-BR transmembrane β-barrel with the protruding surface exposed loop (Epitope CETPRIRGTGTRELE corresponding to amino acid residues 39–53) generated using PRED TMBB. Extracellular N-terminus.

Wavelength:220 nm
<<LC Time Program>>

Time	Module	Command	Value
0.01	Pumps	Solvent B Conc.	5
25.00	Pumps	Solvent B Conc.	65
25.01	Pumps	Solvent B Conc.	95
27.00	Pumps	Solvent B Conc.	95
27.01	Pumps	Solvent B Conc.	5
33.00	Pumps	Solvent B Conc.	5
33.01	Controller	Stop	

<<Column Performance>>
<Detector A>
Column : Inertsil ODS-3 4.6 x 250 mm
Equipment:ZJ17010508

<Chromatogram>

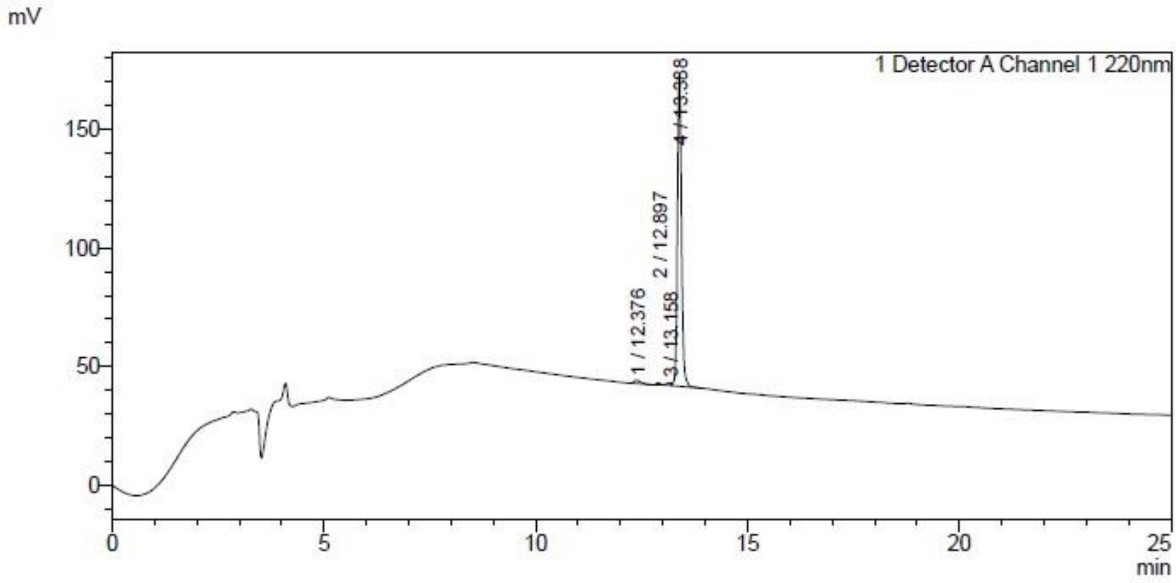

<Peak Table>

Detector A Channel 1 220nm

Peak#	Ret. Time	Area	Height	Area%
1	12.376	15899	1428	1.759
2	12.897	5164	877	0.571
3	13.158	8655	1077	0.957
4	13.388	874374	130416	96.713
Total		904091	133798	100.000

Scheme S1B. HPLC analysis of mouse CCK-B peptide. Reversed-phase high performance liquid chromatography (RP-HPLC) to analyze the purity of target peptide.

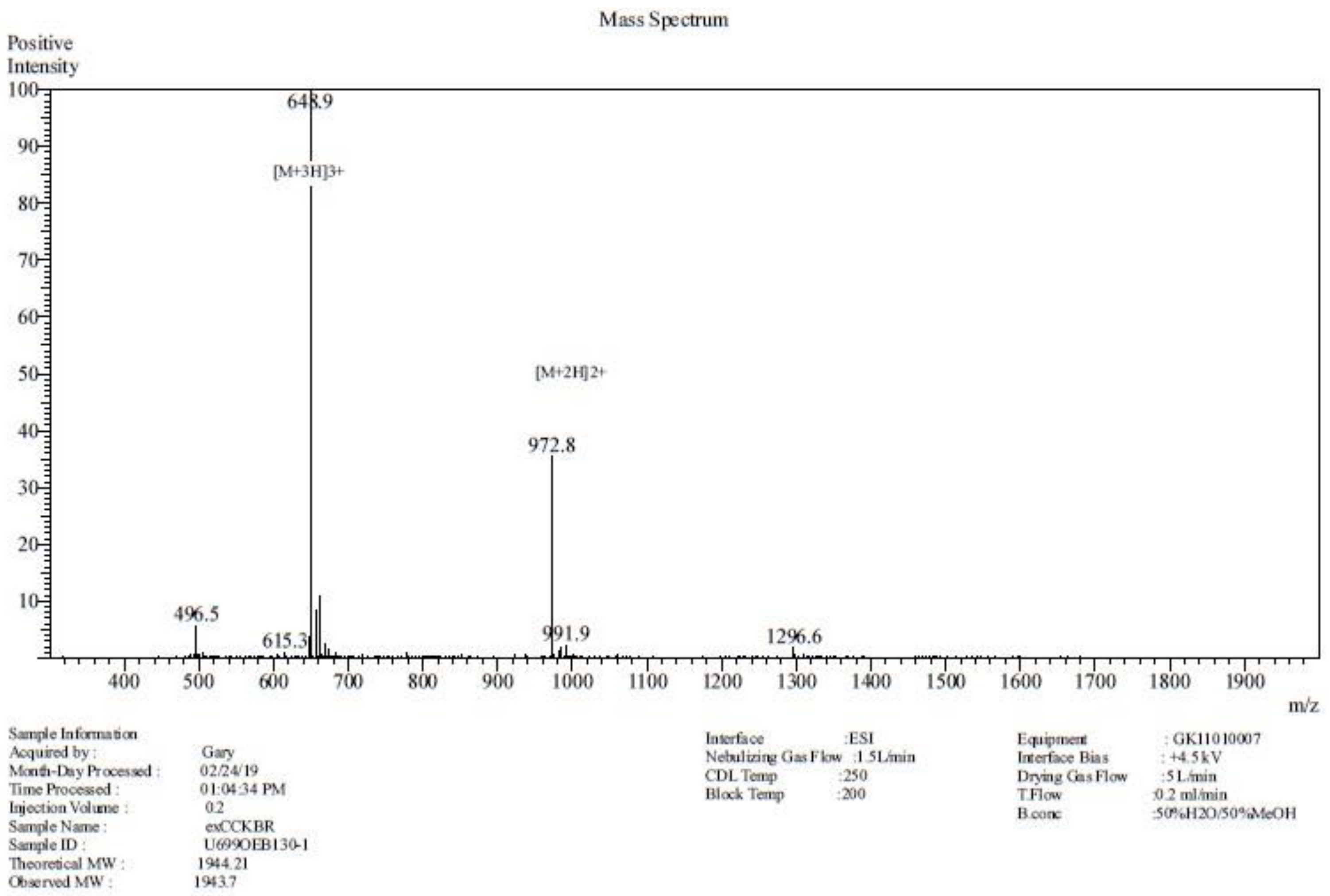

Scheme S1C. MS analysis of mouse CCK-B peptide. Electrospray ionization mass spectrometry (ESI-MS) analysis was used to confirm the molecular weight of the target peptide.

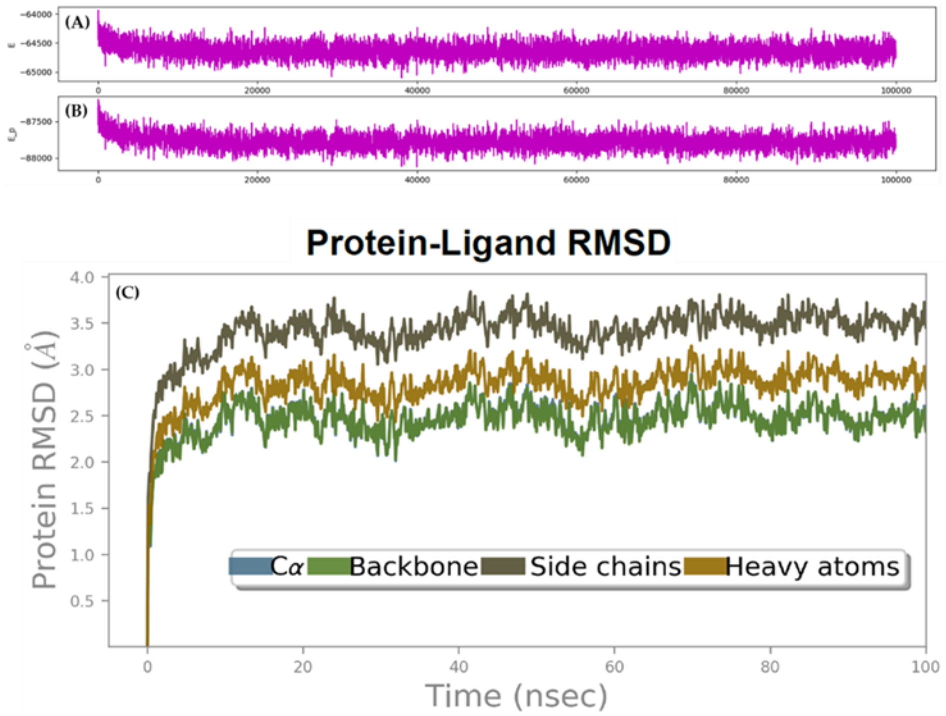

Scheme S2. Results of a 100 ns MD simulation. Total energy (E), potential energy (E_p) and root mean square deviations (RMSD) as functions of scFv77-2 and CCK-B peptide molecular dynamics simulation time. (**A**) Total energy graph of scFv77-2 and CCK-B peptide complex. (**B**) Potential energy graph of scFv77-2 and CCK-B peptide complex. (**C**) RMSD graph of scFv77-2 and CCK-B peptide complex.

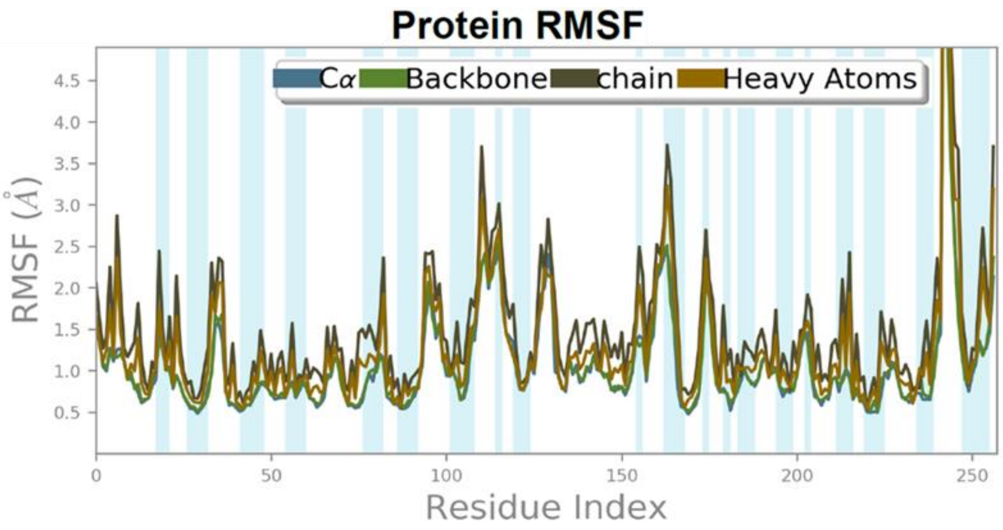

Scheme S3. Molecular dynamics simulation analysis of scFv77-2 and CCK-B peptide complex. Image represents the RMSF and residues fluctuation graph of scFv77-2-CCK-B peptide complex. Peaks indicate areas of the protein that fluctuate most during the simulation. Alpha helical and beta strand regions are highlighted in red and blue backgrounds, respectively. These regions are defined by helices or strands that persist over 70% of the entire simulation. The RMSF of the protein correlated with the experimental X-ray B-factor (right *y*-axis).

Supplementary Tables

Table S1. Nucleotide sequences of primers used.

Primer Name	Primer Sequence (5′ – 3′)
	**VH Primers**
MVH_F1	CGAGAAGACCGGCAGCGGTGGGGCAGAGCTTGTGAAGCCA
MVH_F2	CGAGAAGACCGGCAGCGGTGGAGGAGGCTTGATGCAACCT
MVH_F3	CGAGAAGACCGGCAGCGGTGGACCTGAGCTGGAGATGCCT
MVH_F4	CGAGAAGACCGGCAGCGGTGGACCTGGCCTGGTGAGACCT
MVH_F5	CGAGAAGACCGGCAGCGGTGGGGGAGGCTTAGTGAAGCCT
MVH_F6	CGAGAAGACCGGCAGCGGTGGGGCAGAGCTTGTGAAGCCA
MVH_F7	CGAGAAGACCGGCAGCGGTGGAGGGGGCTTGGTACAGCCT
MVH_F8	CGAGAAGACCGGCAGCGGTGGGGCAGAGCTTGTGAGGTCA
MVH_F9	CGAGAAGACCGGCAGCGGT GAKGTRMAGCTTCAGGAGTC
MVH_F10	CGAGAAGACCGGCAGCGGT GAGGTBCAGCTBCAGCAGTC
MVH_F11	CGAGAAGACCGGCAGCGGT CAGGTGCAGCTGAAGSASTC
MVH_F12	CGAGAAGACCGGCAGCGGT GAGGTCCARCTGCAACARTC
MVH_F13	CGAGAAGACCGGCAGCGGT CAGGTYCAGCTBCAGCARTC
MVH_F14	CGAGAAGACCGGCAGCGGT CAGGTYCARCTGCAGCAGTC
MVH_F15	CGAGAAGACCGGCAGCGGT CAGGTCCAGGTGAAGCAGTC
MVH_F16	CGAGAAGACCGGCAGCGGT GAGGTGAASSTGGTGGAATC
MVH_F17	CGAGAAGACCGGCAGCGGT GAVGTGAWGYTGGTGGAGTC
MVH_F18	CGAGAAGACCGGCAGCGGT GAGGTGCAGSKGGTGGAGTC
MVH_F19	CGAGAAGACCGGCAGCGGT GAKGTGCAMCTGGTGGAGTC
MVH_F20	CGAGAAGACCGGCAGCGGT GAGGTGAAGCTGATGGARTC
MVH_F21	CGAGAAGACCGGCAGC GGT GAGGTGCARCTTGTTGAGTC
MVH_F22	CGAGAAGACCGGCAGCGGT GARGTRAAGCTTCTCGAGTC
MVH_F23	CGAGAAGACCGGCAGCGGT GAAGTGAARSTTGAGGAGTC
MVH_F24	CGAGAAGACCGGCAGCGGT CAGGTTACTCTRAAAGWGTSTG
MVH_F25	CGAGAAGACCGGCAGCGGT CAGGTCCAACTVCAGCARCC
MVH_F26	CGAGAAGACCGGCAGCGGT GATGTGAACTTGGAAGTGTC
MVH_F27	C GAG AAG ACC GGC AGC GGT GAGGTGAAGGTCATCGAGTC
MVH_R1	GGAGCCGCCGCCGCCGCCAGAACCACCACCACCGGATCCACCACCACCCGAGGAAACGGTGACCGTGGT
MVH_R2	GGAGCCGCCGCCGCCGCCAGAACCACCACCACC**GGATCC**ACCACCACCCGAGGAGACTGTGAGAGTGGT
MVH_R3	GGAGCCGCCGCCGCCGCCAGAACCACCACCACC**GGATCC**ACCACCACCCGCAGAGACAGTGACCAGAGT
MVH_R4	GGAGCCGCCGCCGCCGCCAGAACCACCACCACC**GGATCC**ACCACCACCCGAGGAGACGGTGACTGAGGT
	**VL Primers**
MVL_F1	GGCGGCGGCGGCTCCGGTGGTGGTGGATCCGCAATCATGTCTGCATCTCC
MVL_F2	GGCGGCGGCGGCTCCGGTGGTGGTGGATCCGCCTCCCTATCTGTATCTGTG
MVL_F3	GGCGGCGGCGGCTCCGGTGGTGGTGGATCCGCCTCCCTATCTGCATCTGTG
MVL_F4	GGCGGCGGCGGCTCCGGTGGTGGTGGATCCCTCACTTTGTCGGTTACCATT
MVL_F5	GGCGGCGGCGGCTCCGGTGGTGGTGGATCCTCAGCCTCTTTCTCCCTGGGA
MVL_F6	GGCGGCGGCGGCTCCGGTGGTGGTGGATCCTCCTCCCTGAGTGTGTCAGCA
MVL_F7	GGCGGCGGCGGCTCCGGTGGTGGTGGATCCCTCTCCCTGCCTGTCAGTCTT
MVL_F8	GGCGGCGGCGGCTCCGGTGGTGGTGGATCCCTCTCCCTGCCTGTCAGTCTT
MVL_R1	AGTGCGGCCGCATCAGCCCGTTTTATTTCCAG
MVL_R2	AGTGCGGCCGCATCAGCCCGTTTTATTTCCAA
MVL_R3	AGTGCGGCCGCACCTAGGACAGTGACCTTGGT
MVL_R4	AGTGCGGCCGCATCAGCCCGTTTCAGGTCCAG
	**T7 Promoter**
RDT7	CTATAGAAGGGTAATACGACTCACTATAGGGCGAATTCCACCATGGCC
T7	TAATACGACTCACTATAGGG
SP6	ATTTAGGTGACACTATAG

Table S2. Analysis of VH and VL gene usage in the selected scFv clones.

Clone	VH						VL				
	**V- seg alignment**	**D-seg alignment**	**J- seg alignment**	**Name**	**Differences from germline (nucleotides, amino acids)[a]**	**VHCDR3**	**V- seg alignment**	**J- seg alignment**	**Name**	**Differences from germline (nucleotides, amino acids)[a]**	**VLCDR3**
8-3	V segment of th Igh-VJ558 VH1 family	DFL16.1, DFL16.1inv, DFL16.1e	JH2 mouse, JH4 mouse, JH1 mouse	musIGHV532	(4, 2)	ARDYGSSFDY	V segment of the IGKV2 subgroup	jk1, jk2, jk4	musIGKV097	(22, 9)	WQGTHFPWT
11-2	V segment of th Igh-VJ558 VH1 family	DFL16.2, DST4a, DST4b	JH4 mouse, JH2 mouse, JH1 mouse	musIGHV559	(21, 11)	ARAATWYYAM DY	V segment of the IGKV4/5 subgroup	Jk1, jk2, jk4	musIGKV082	(30, 13)	HQWSSYPWT
13-3	V segment of th Igh-VJ558 VH1 family	DFL16.2, DFL16.1, DST4.3	JH1 mouse, JH2 mouse, JH1B6 mouse	musIGHV532	(27, 9)	AGDNYGYWYFD V	V segment of the IGKV8 subgroup	Jk1, jk2, jk4	musIGKV069	(21, 8)	QNDHSYPYT
14-3	V segment of th Igh-V15 VH15 family	PseudoD2in v, IGHD6-2*02inv, DFL16.2	JH3 mouse, JH2 mouse, JH1 mouse	musIGHV154	(18, 8)	NAGGRFAY	V segment of the IGKV8 subgroup	jk1, jk2, jk4	musIGKV069	(21, 8)	QNDHSYPYT

77-2	V segment of th Igh-V15 VH15 family	PseudoD2inv, IGHD6-2*02inv, DFL16.2	JH3 mouse, JH2 mouse, JH1 mouse	musIGHV154	(22, 9)	NAGGRFAY	V segment of the IGKV8 subgroup	Jk2, jk4, jk1	musIGKV069	(21, 8)	QNDHSYPYT
93-1	V segment of th Igh-VQ52 VH2 family	DFL16.1, P5, DSP2.13	JH4 mouse, JH2 mouse, JH1 mouse	musIGHV171	(2, 2)	ARGLRGAMDY	V segment of the IGKV8 subgroup	Jk4, jk2, jk5	musIGKV184	(39, 17)	QNDHSYPFT
134-1	V segment of th Igh-VJ558 VH1 family	DSP2.9, PseudoD3, DSP2.6	JH4 mouse, JH2 mouse, JH1 mouse	musIGHV707	(24, 10)	ARSEGYLPFDY	V segment of the IGKV2 subgroup	jk1, jk2, jk5	musIGKV145	(19, 8)	LQGTHQPRT

[a] Nucleotide and amino acid differences in V-gene segment, excluding CDR3. The nucleotide sequences were analyzed using V-BASE2 (VBASE2 - the integrative germ-line V gene database) (http://www.vbase2.org/vbscAb.php).
